# The feasibility and safety of his-purkinje conduction system pacing in patients with heart failure with severely reduced ejection fraction

**DOI:** 10.3389/fcvm.2023.1187169

**Published:** 2023-05-22

**Authors:** Chengming Ma, Zhongzhen Wang, Zhulin Ma, Peipei Ma, Shiyu Dai, Nan Wang, Yiheng Yang, Guocao Li, Lianjun Gao, Yunlong Xia, Xianjie Xiao, Yingxue Dong

**Affiliations:** ^1^ Department of Cardiology, Institute of Cardiovascular Diseases, First Affiliated Hospital of Dalian Medical University, Dalian, China; ^2^ Department of Graduate School, Dalian Medical University, Dalian, China

**Keywords:** his-purkinje conduction system pacing, heart failure, severely reduced ejection fraction, his-bundle pacing, left bundle branch pacing

## Abstract

**Objective:**

The purpose of this study was to evaluate the feasibility and outcomes of conduction system pacing (CSP) in patients with heart failure (HF) who had a severely reduced left ventricular ejection fraction (LVEF) of less than 30% (HFsrEF).

**Methods:**

Between January 2018 and December 2020, all consecutive HF patients with LVEF < 30% who underwent CSP at our center were evaluated. Clinical outcomes and echocardiographic data [LVEF and left ventricular end-systolic volume (LVESV)], and complications were all recorded. In addition, clinical and echocardiographic (≥5% improvement in LVEF or ≥15% decrease in LVESV) responses were assessed. The patients were classified into a complete left bundle branch block (CLBBB) morphology group and a non-CLBBB morphology group according to the baseline QRS configuration.

**Results:**

Seventy patients (66 ± 8.84 years; 55.7% male) with a mean LVEF of 23.2 ± 3.23%, LVEDd of 67.33 ± 7.47 mm and LVESV of 212.08 ± 39.74 ml were included. QRS configuration at baseline was CLBBB in 67.1% (47/70) of patients and non-CLBBB in 32.9%. At implantation, the CSP threshold was 0.6 ± 0.3 V @ 0.4 ms and remained stable during a mean follow-up of 23.43 ± 11.44 months. CSP resulted in significant LVEF improvement from 23.2 ± 3.23% to 34.93 ± 10.34% (*P* < 0.001) and significant QRS narrowing from 154.99 ± 34.42 to 130.81 ± 25.18 ms (*P* < 0.001). Clinical and echocardiographic responses were observed in 91.4% (64/70) and 77.1% (54/70) of patients. Super-response to CSP (≥15% improvement in LVEF or ≥30% decrease in LVESV) was observed in 52.9% (37/70) of patients. One patient died due to acute HF and following severe metabolic disorders. Baseline BNP (odds ratio: 0.969; 95% confidence interval: 0.939–0.989; *P* = 0.045) was associated with echocardiographic response. The proportions of clinical and echocardiographic responses in the CLBBB group were higher than those in the non-CLBBB group but without significant statistical differences.

**Conclusions:**

CSP is feasible and safe in patients with HFsrEF. CSP is associated with a significant improvement in clinical and echocardiographic outcomes, even for patients with non-CLBBB widened QRS.

## Introduction

Heart failure (HF) remains a major health and economic burden worldwide with high incidence, hospitalizations, and mortality (1, 2). HF patients with a severely reduced left ventricular ejection fraction (LVEF) of less than 30% (HFsrEF) are not rare. These patients have a high risk of admission, progression to advanced HF, and mortality even after receiving maximal and optimal pharmacological therapy. Patients with HFsrEF are becoming more prevalent due to the ageing population, increasing number of HF patients, and improved treatment (3). Providing a better treatment is crucial to improving prognosis and lowing medical costs for these patients.

Conduction system pacing (CSP), including His bundle pacing (HBP) and left bundle branch pacing (LBBP), is an alternative strategy for achieving cardiac resynchronization therapy (CRT) in HF patients with reduced EF (HFrEF) and ventricular desynchrony (4, 5). Most guidelines recommended LVEF ≤ 35% as a crucial inclusion criterion for CRT selection, and the LVEF values in most randomized controlled trials (RCTs) focusing on CSP ranged from 30% to 40%. However, due to limited clinical trials, the feasibility, safety and benefits of CSP in patients with HFsrEF (<30%) remains unknown. Furthermore, patients with typical complete left bundle branch block (CLBBB) QRS morphology show a better CRT response than patients with non-CLBBB morphology (6). However, CRT response in patients with a non-CLBBB widened QRS remains uncertain.

This research aimed to describe the feasibility and safety of CSP in patients with HFsrEF and evaluate the clinical and echocardiographic responses to CSP.

## Materials and methods

This was a retrospective, single-center and observational study. Written informed consent was obtained from all enrolled patients. The local ethics committee approved this study. The data from our research are available from the corresponding author upon request.

### Study patients

**Figure 1:**
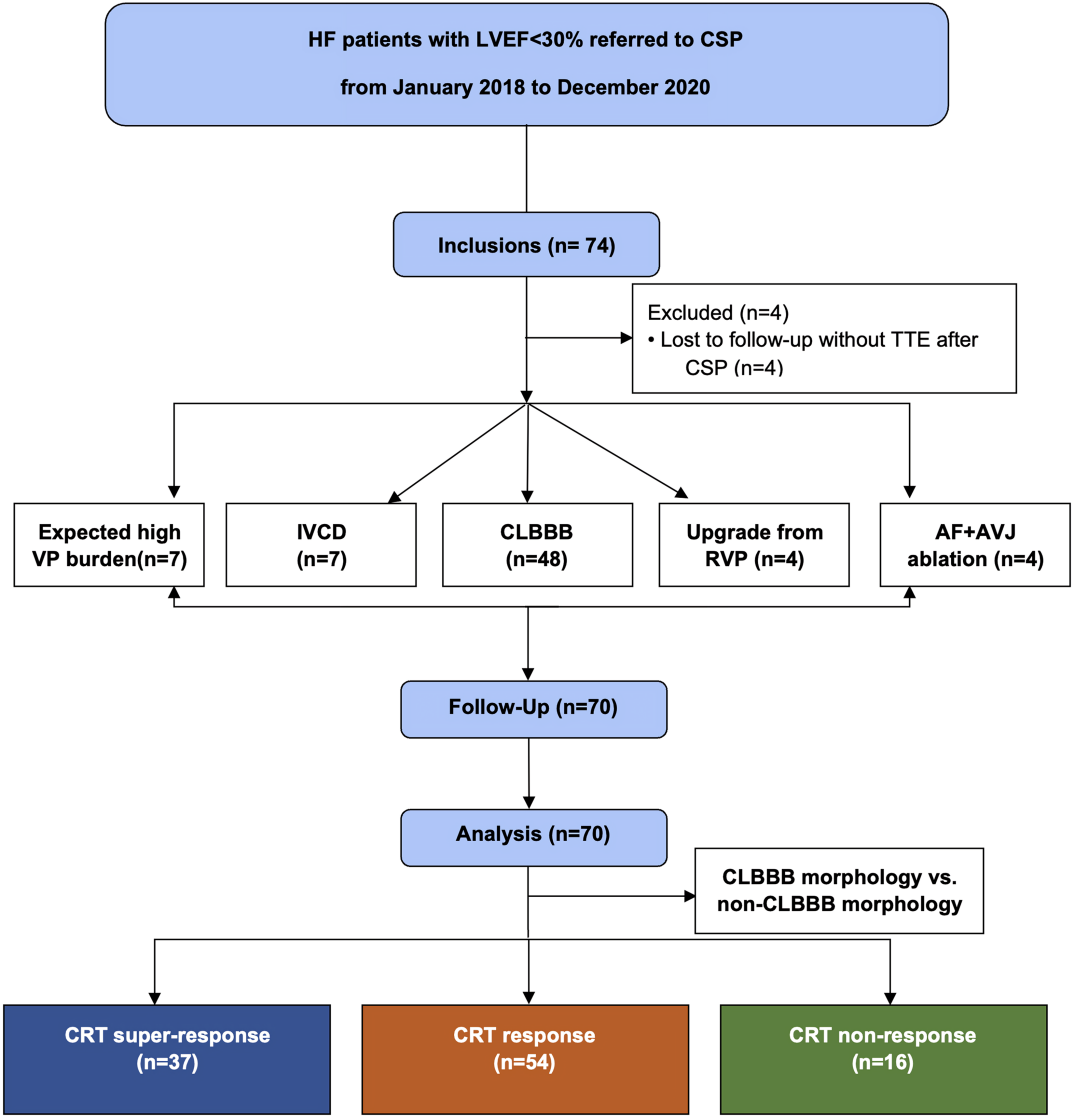
Flow chart of patients screened for inclusion in this study. IVCD, inner-ventricular conduct delay; CRT, cardiac resynchronization therapy; VP, ventricular pacing; CSP, conduction system pacing; CLBBB, complete left branch bundle block; RVP, right ventricular pacing; AF, atrial fibrillation; AVJ, atrioventricular junction; TTE, transthoracic echocardiography.

All consecutive HF patients with LVEF < 30% treated with CSP in our center from January 2018 to December 2020 were collected, and all the patients met the CRT indication (Figure 1). All patients received guideline-directed medical treatment for at least three months before implantation. Patients who lost follow-up or could not perform device programming after the CSP procedure were excluded from this study.

### Implant procedure

The detailed implant procedure was found in this article (7). In brief, a pre-shaped sheathing canal was inserted into the superior vena cava via the left or right subclavian vein approach. A Select Secure pacing lead (Model 3,830–69 cm, Medtronic, Minneapolis, Minnesota, USA) was introduced into the right atrium via a fixed curve sheathing canal (C315HIS; Medtronic, Minneapolis, Minnesota, USA). Endocardial mapping was performed using a unipolar mapping technique with the pacing lead. During the HBP procedure, the pacing lead initially obtained the HB potential. The preferred site was defined as a narrow paced QRS with the same QRS morphology as the intrinsic QRS and successful correction of LBBB with satisfactory pacing thresholds. During the LBBP procedure, the initial site of LBBP at the right ventricular septum was advanced 1–2 cm anteriorly and inferiorly into the ventricle along an imaginary line between the His and the right ventricle apex in the right anterior oblique projection. The location of the LBBP site was guided using the distal HBP location or the paced QRS morphology (“W” pattern with a notch at the nadir of the QRS in lead V1). Subsequently, the lead was inserted deeply into the muscular interventricular septum with caution. When LBB capture was confirmed with a low threshold, additional rotations were stopped. The preferred location was determined using criteria that had already been disclosed. An abrupt reduction in the stim to LV active time (LVAT) of longer than 10 ms and the morphologies of qR, Qr, or rSR′ in lead V1 was the criteria for the LBBB correct in the LBBP procedure.

### Follow-up

All patients were followed up at the clinic 1-month, 6-month, and 12-month following the CSP procedure and then every 6 months after that. The device programming was performed at the time of discharge and each subsequent visit. The pacing parameters were collected, including bipolar R-wave amplitude, bipolar capture threshold and impedance. All transthoracic echocardiography (TTE) parameters at pre-implantation and post-implantation were collected, including LVEF, left ventricular end-diastolic diameter (LVEDd), left ventricular end-systolic volume (LVESV), and emerging or worsening tricuspid regurgitation (TR). Post-implantation echocardiographic outcomes were based on the last available follow-up. The baseline and post-procedural ECGs were analyzed, including QRS duration, QRS pattern, and QRS axis. The New York Heart Association (NYHA) cardiac function classification was documented. The following complications were recorded: a significant increase in pacing threshold (defined as a >1 V increase in capture threshold after implantation or capture threshold >5 V @ 0.4 ms at any follow-up visit), loss of capture, lead dislodgement, and cardiac perforation. All patients received guideline-directed medical treatment.

### Response to CSP

The primary outcome was the clinical and echocardiographic responses to CSP. The secondary outcome was rehospitalization for HF and all-cause death. The definition of response to CSP was consistent with most studies in the literature, including the following: (1) the documentation of an increase in LVEF ≥ 5% or decrease in LVESV ≥ 15% after 6 months relative to baseline, (2) clinical improvement in NYHA class ≥1 or NHYA class Ⅰ at last observation carried forward, or a ≥25% increase in 6-MWD (8–10). The super-response to CSP was defined as a ≥15% improvement in the LVEF or a ≥30% decrease in the LVESV with clinical improvements after 6 months relative to baseline. A non-responder was classified as a patient who had worsened HF, no improvement in clinical features, or a <5% increase in LVEF.

### CLBBB morphology vs. non-CLBBB morphology

According to the baseline QRS configuration, the patients were classified into a CLBBB morphology (CLBBB + RVP) group and a non-CLBBB morphology group. RVP causes an CLBBB type QRS pattern and results in LV desynchrony; thus, patients who upgraded from RVP were assigned to the CLBBB morphology group. The clinical and echocardiographic responses to CSP were compared.

### Statistical analysis

The data analysis was performed using SPSS 22.0 (SPSS Inc., Chicago, USA). The quantitative data were expressed as the mean ± standard deviation (SD) if normally distributed; they were described by median [25th quarter, 75th quarter] if non-normally distributed. The homogeneity of variance was tested by Kolmogorov–Smirnov Goodness. The categorical data were expressed in terms of frequency and percentage. An unpaired t-test or nonparametric Mann–Whitney *U* test was performed for comparison between groups for quantitative data. For categorical variables, chi-square tests or Fisher's exact tests were used. A 2-tailed *P*–value <0.05 was considered statistically significant.

## Results

### Baseline characteristics of study patients

Between January 2018 to December 2021, 70 patients (66 ± 8.84 years; 55.7% male) were included in this study. The mean baseline LVEF of these patients was 23.2 ± 3.23%, and their mean baseline LVEDd and LVESV were 67.33 ± 7.47 mm, and 212.08 ± 39.74 ml, respectively. The mean NYHA class was 3.46 ± 0.55 (NYHA Ⅱ in 2 (2.9%) patients, Ⅲ in 33 (47.1%) patients, Ⅲ-Ⅳ in 1 (1.4%) patient, and Ⅳ in 34 (48.6%) patients) with a baseline BNP of 787.63 pg/ml. The mean QRS duration was154.99 ± 34.42 ms. Among them, CRT indicated for HFrEF with CLBBB in 48 (68.6%) patients, HFrEF with inner-ventricular conduct delay (IVCD) in 7 (10%) patients, HFrEF with permanent atrial fibrillation (AF) being eligible for atrioventricular junction (AVJ) ablation in 4 (5.7%) patients, expected high ventricular pacing (VP) burden in 7 (10%) patients and upgrading for low LVEF due to RV pacing in the remaining 4 (5.7%) patients (Figure 1). Other baseline characteristics of the enrolled patients are shown in Table 1.

**Table 1:** Baseline characteristics of enrolled patients.

	*n *= 70
Age (years), mean ± SD	66 ± 8.84
Male, *n* (%)	39 (55.7%)
ICM, *n* (%)	18 (25.7%)
Hypertension, *n* (%)	32 (45.7%)
Diabetes mellitus, *n* (%)	18 (25.7%)
DCM, *n* (%)	30 (42.9%)
Atrial fibrillation, *n* (%)	30 (42.9%)
CKD, *n* (%)	7 (10%)
Valve replacement, *n* (%)	9 (12.9%)
Mitral valve	5 (7.1%)
Aortic valve	3 (4.3%)
Mitral valve replacement + tricuspid annuloplasty	1 (1.4%)
Tricuspid regurgitation	43 (61.4%)
NYHA class	3.46 ± 0.55
Ⅰ	0
Ⅱ	2 (2.9%)
Ⅲ	33 (47.1%)
Ⅲ–Ⅳ	1 (1.4%)
Ⅳ	34 (48.6%)
6-MWD (m), mean ± SD	166.14 ± 54.52
BNP (pg/ml), median, [25% percentile,75% percentile] IQR1854	787.63 [402.83, 1686.63]
LVEF (%), mean ± SD	23.2 ± 3.23
LVEDd (mm), mean ± SD	67.33 ± 7.47
LVESV (ml), mean ± SD	212.08 ± 39.74
QRS duration (ms), mean ± SD	154.99 ± 34.42
QRS axis (°), median, [25% percentile,75% percentile] IQR75	−7, [−33.25, 35.5]
CLBBB	48 (68.6%)
IVCD	7 (10%)
Expected high VP burden	7 (10%)
Upgrade from RVP	4 (5.7%)
AF + AVJ ablation	4 (5.7%)
Previous implanted pacemakers, *n* (%)	14 (20%)
CRT	13 (16.5%)
RVP	4 (5.7%)
Pharmacological therapy for HF	
ACE inhibitors/ARB/ARNI	25 (35.7%)
Beta blockers	39 (55.7%)
MRA	60 (85.7%)
Diuretics	54 (77.1%)

DCM, dilated cardiomyopathy; ICM, Ischemic cardiomyopathy; CKD, chronic kidney disease; 6-MWD, 6 min’ walk distance; BNP, brain natriuretic peptide; LVEF, left ventricular ejection fraction; LVEDd, left ventricular end-diastolic diameter; LVESV, left ventricular end-systolic volume; NYHA, New York Heart Association; CLBBB, complete left branch bundle block; IVCD, inner-ventricular conduct delay; CRT, cardiac resynchronization therapy; RVP, right ventricular pacing; MRA, mineralocorticoid receptor antagonists; ACE, angiotensin-converting enzyme; ARB, angiotensin receptor blockers; ARNI, angiotensin receptor neprilysin inhibitors; AVJ, atrioventricular junction.

### CSP implantation

The CSP procedure was successfully performed in all patients. Among them, 52 (74.29%) patients received HBP, and 18 (25.71%) patients received LBBP. After the implantation, all patients with widened QRS (CLBBB + RVP) were corrected, and the mean postprocedural QRS duration was 131.75 ± 22.29 ms, significantly narrower than baseline (166.52 ± 23.99 ms, *P* < 0.001).

The mean capture threshold at implantation was 0.63 ± 0.31 V@ 0.4 ms, slightly decreased at 1-month post-implantation, and no increase was observed during the 1-year follow-up period. The R-wave amplitudes were 4.10 [2.73, 7.07] mV and impedance were 635.90 ± 141.73 Ω, with no significant increase noted during the follow-up. Capture threshold increase >1 V was noted in 4 (5.71%) patients. No dislodgments were observed. Other procedure related complications, such as thrombosis, infection, perforation, and stroke, were also not detected during the follow-up.

### Clinical outcomes

**Figure 2:**
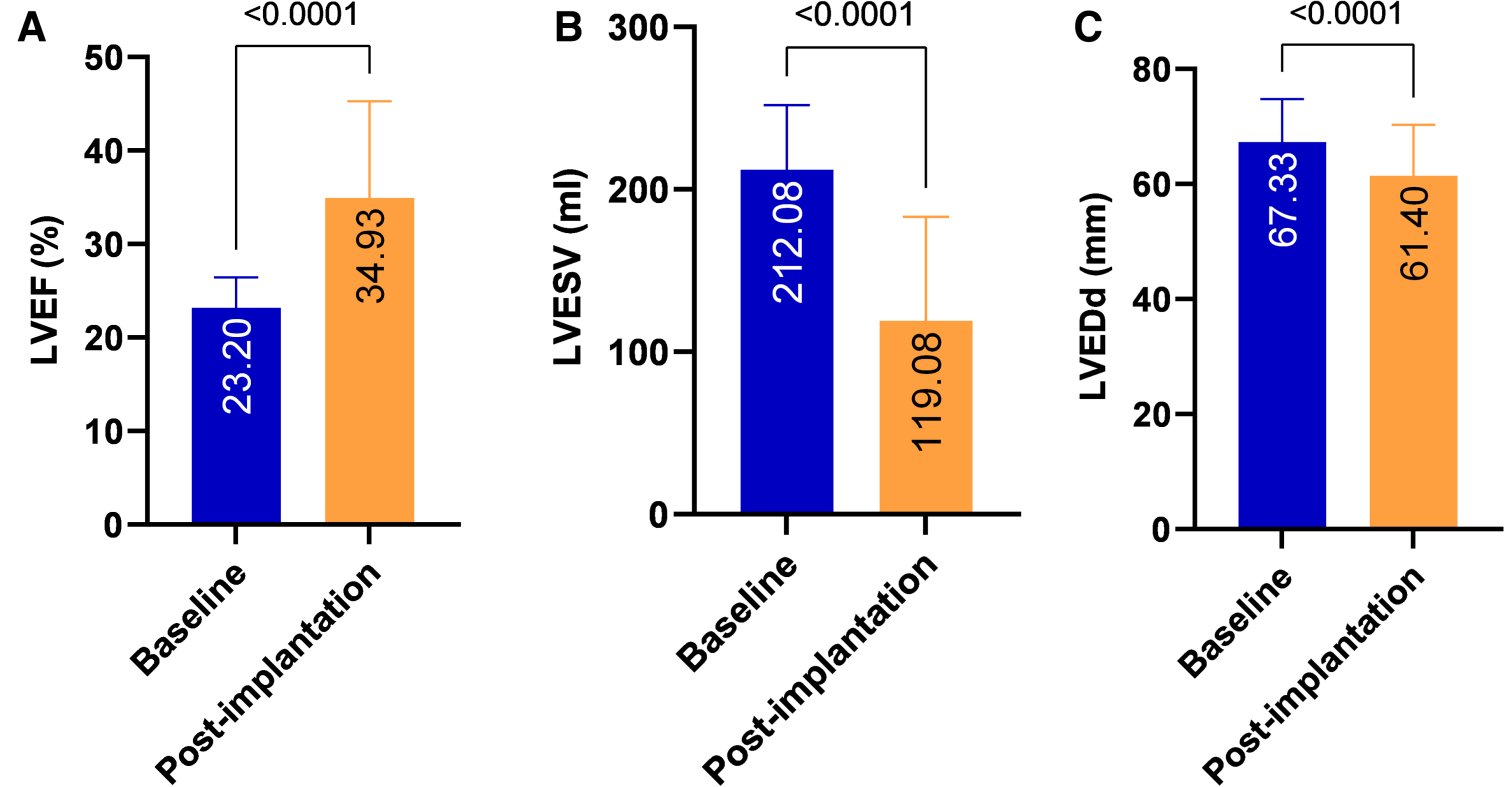
Echocardiographic performance after CSP. Significant improvements in LVEF (A), LVESV (B) and LVEDd (C) were observed after CSP in all patients. LVEF, left ventricular ejection fraction; LVEDd, left ventricular end-diastolic diameter; LVESV, left ventricular end-systolic volume; CSP, conduction system pacing.

**Figure 3:**
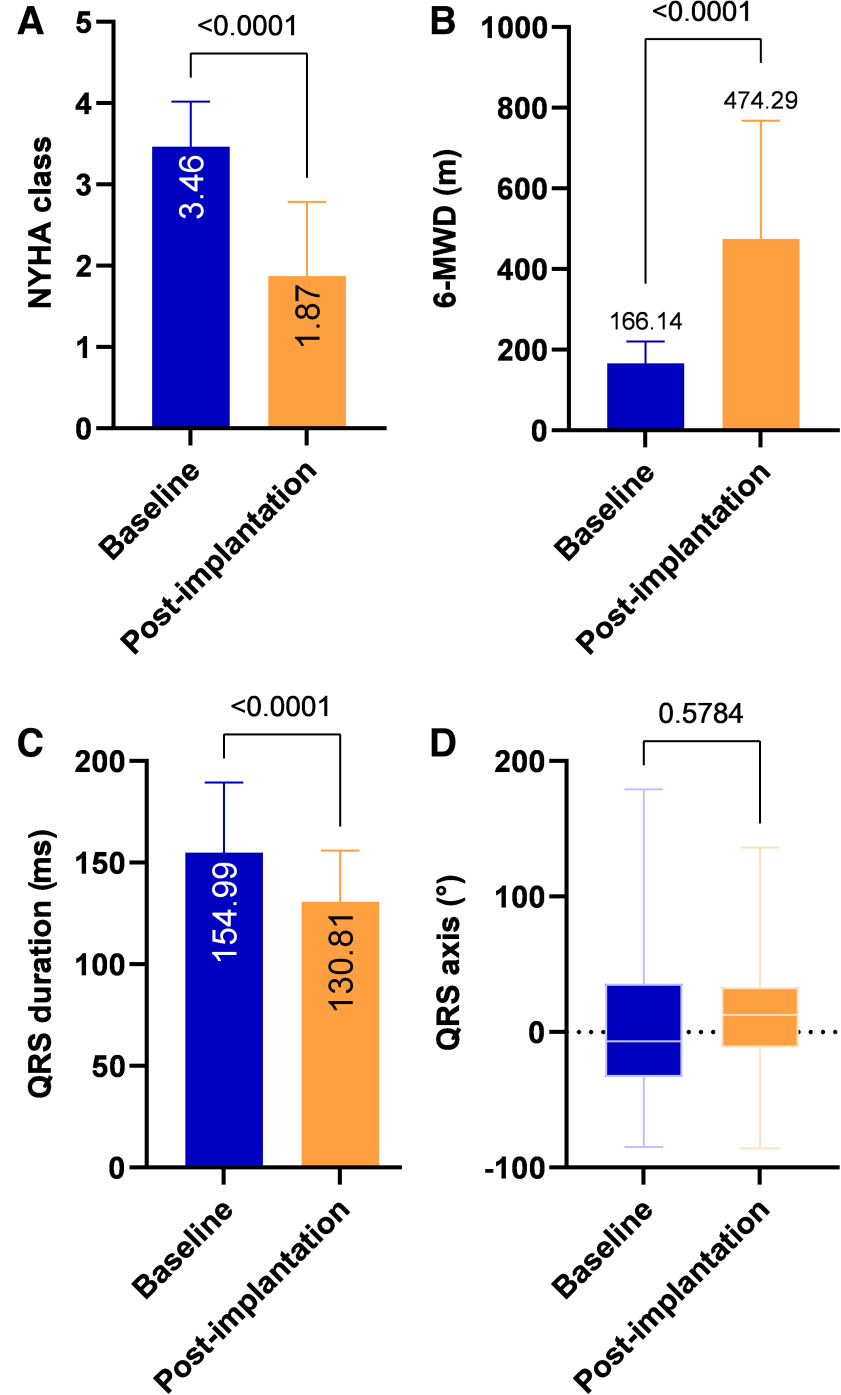
Clinical outcomes after CSP. Compared with pre-implantation, NYHA class and 6-MWD improved significantly at post-implantation (A, B). QRS duration after CSP was significantly reduced (C) with no apparent change in QRS axis (D). 6-MWD, 6-minute walk distance; NYHA, New York Heart Association.

During the mean follow-up period of 23.43 ± 11.44 months, compared with pre-implantation, a significant increase in LVEF (23.2 ± 3.23% vs. 034.93 ± 10.34%, *P* < 0.001), a decrease in LVESV (212.08 ± 39.74 vs. 119.08 ± 64.09 ml, *P* < 0.001) and a reduction in LVEDd (67.33 ± 7.47 vs. 61.4 ± 8.9 mm, *P* < 0.001) were observed (Table 2 and Figure 2). A significant improvement in the NYHA class and 6-MWD post-implantation was observed in all patients (3.46 ± 0.55 vs. 1.87 ± 0.92, *P* < 0.001; 166.14 ± 54.52 m vs. 474.29 ± 293.38 m, *P* < 0.001, Table 2 and Figure 3). A significant QRS narrowing from 154.99 ± 34.42 to 130.81 ± 25.18 ms (*P* < 0.001) was observed after the CSP (Figure 3). In addition, the mean number of rehospitalizations was 0.53 ± 0.28, and one patient died 13 months after implantation due to acute HF and subsequently severe metabolic disorders.

**Table 2:** Clinical outcomes of the patients and changes in TTE.

	Baseline	Post-implantation	*P*
NYHA classification	3.46 ± 0.55	1.87 ± 0.92	<0.001
None	0	8 (11.4%)	
Ⅰ	0	10 (14.3%)	
Ⅱ	2 (2.9%)	35 (50%)	
Ⅲ	33 (47.1%)	17 (24.3%)	
Ⅲ–Ⅳ	1 (1.4%)	0	
Ⅳ	34 (48.6%)	0	
6-MWD (m)	166.14 ± 54.52	474.29 ± 293.38	<0.001
LVEF (%)	23.2 ± 3.23	34.93 ± 10.34	<0.001
LVEDd (mm)	67.33 ± 7.47	61.4 ± 8.9	<0.001
LVESV (ml)	212.08 ± 39.74	119.08 ± 64.09	<0.001
QRS duration (ms)	154.99 ± 34.42	130.81 ± 25.18	<0.001
QRS axis (°)	−7, [−33.25, 35.5]	12.5, [−11.5, 33]	0.578

LVEF, left ventricular ejection fraction; LVEDd, left ventricular end-diastolic diameter; LVESV, left ventricular end-systolic volume; NYHA, New York Heart Association.

**Table 3:** Independent predictors of CSP response.

Variables	Univariate analysis		Multivariable analysis	
	OR (95%CI)	*P*	OR (95%CI)	*P*
Sex (male)	0.696 (0.222–2.187)	0.535	0.56 (0.101–3.103)	0.507
Age	1.007 (0.946–1.073)	0.82	1.07 (0.974v1.175)	0.158
CLBBB	1.847 (0.586–5.819)	0.295	3.174 (0.303–33.31)	0.335
ICM	0.476 (0.144–1.578)	0.225	0.272 (0.036–2.055)	0.207
Hypertension	2.2 (0.673–7.189)	0.192	10.274 (1.176–13.531)	0.083
Diabetes mellitus	1.05 (0.291–3.795)	0.941	0.862 (0.147–5.044)	0.869
DCM	0.954 (0.31–2.939)	0.935	4.72 (0.621–6.220)	0.134
AF	0.688 (0.224–2.108)	0.512	0.870 (0.155–4.874)	0.874
CKD	0.347 (0.069–1.746)	0.199	0.173 (0.023–1.316)	0.090
LVEF	1.002 (0.842–1.192)	0.986	0.942 (0.727–1.22)	0.648
LVEDd	0.947 (0.876–1.025)	0.176	0.992 (0.872–1.129)	0.907
LVESV	0.355 (0.068–1.841)	0.218	1.007 (0.975–1.041)	0.666
BNP	0.969 (0.939–0.999)	0.045	0.969 (0.939–0.989)	0.045
NYHA class	1.121 (0.409–3.076)	0.824	1.452 (0.125–16.87)	0.766
6-MWD	1.24 (0.414–3.716)	0.701	1.108 (0.062–19.73)	0.944
QRS duration	1.007 (0.856–1.186)	0.929	1.108(0.874–1.406)	0.397

DCM, dilated cardiomyopathy; ICM, Ischemic cardiomyopathy; CKD, chronic kidney disease; 6-MWD, 6 min walk distance; BNP, brain natriuretic peptide; LVEF, left ventricular ejection fraction; LVEDd, left ventricular end-diastolic diameter; LVESV, left ventricular end-systolic volume; NYHA, New York Heart Association; CLBBB, complete left branch bundle block; IVCD, inner-ventricular conduct delay; CRT, cardiac resynchronization therapy; RVP, right ventricular pacing.

**Figure 4:**
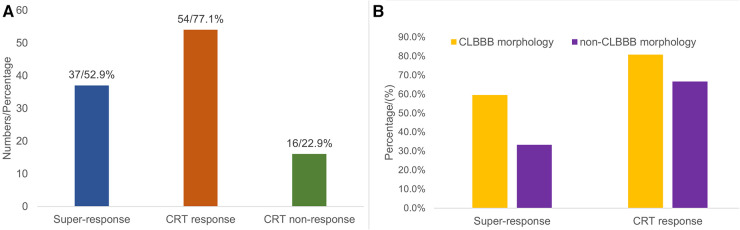
Distribution chart of CRT response. Among all 70 patients, 37 (52.9%) patients were classified as super-responders, 54 (77.1%) patients were classified as CRT-responders, and 16 (22.9%) patients had no response to CRT (A). The proportion of super-response (59.6% vs. 33.3%, *P* = 0.054) and response to CRT (80.8% vs. 66.7%, *P* = 0.219) in the CLBBB group were higher than those in the non-CLBBB group but without significant statistical differences (B). CRT, cardiac resynchronization therapy; CLBBB, complete left branch bundle block.

Of 70 patients in the study, 37 (52.9%) patients achieved a mean increase of 17.78 ± 10.4% in LVEF and were categorized as super-responders (Figure 4A). There were 43 (61.4%) patients who achieved a >10% increase in LVEF. In addition, a positive echocardiographic response to CRT was detected in 54 (77.1%) patients with a mean LVEF increase of 14.61 ± 9.89%. Sixteen (22.9%) patients had a <5% increase in LVEF; however, no patient had a decrease in LVEF during the follow-up. Sixty-four (91.4%) patients obtained a clinical improvement.

After multivariable logistic regression analysis, only baseline BNP (odds ratio: 0.969; 95% confidence interval: 0.939 to 0.989; *P* = 0.045) was associated with CRT response (Table 3).

### CLBBB morphology vs. non-CLBBB morphology

**Figure 5:**
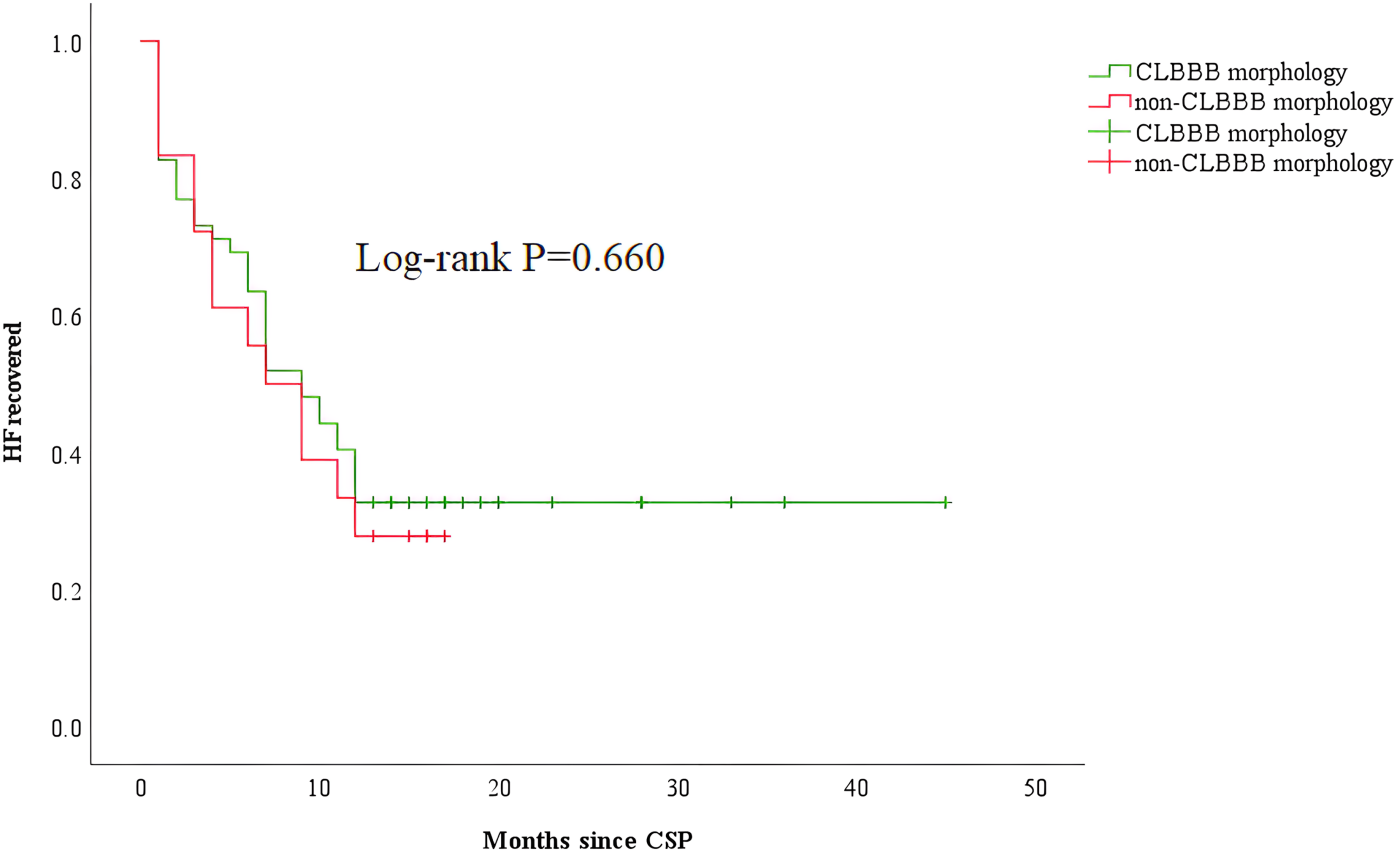
Kaplan–Meier curve comparing HF recovered between patients with CLBBB and non-CLBBB morphology. CSP, conduction system pacing; CLBBB, complete left branch bundle block; HF, heart failure.

Among all the enrolled patients, 52 patients (48 patients with CLBBB and 4 patients upgraded to CSP from RVP) were in the CLBBB morphology group, and 18 patients were in the non-CLBBB morphology. No significant differences in sex, age, HF duration, and baseline BNP were detected between the two groups. The baseline characteristics of the two groups were summarized in Supplementary Table S1. The baseline QRS duration in the CLBBB morphology group was wider than that in the non-CLBBB morphology group. The baseline NYHA class, LVEF, LVEDd, and LVESV in the CLBBB morphology group were more severe than those in the non-CLBBB morphology group. There was no significant statistical difference in post-procedural NYHA class, 6-MWD, LVEF, LVEDd, LVESV, and QRS duration between the two groups (Supplementary Table S2). The proportion of super-response and response to CRT in the CLBBB group were higher than those in the non-CLBBB group but without significant statistical differences (Super-response: 59.6% vs. 33.3%, *P* = 0.054; CRT response: 80.8% vs. 66.7%, *P* = 0.219, Supplementary Table S2 and Figure 4B). No difference in time to HF recovered between the two groups (Figure 5).

## Discussion

The present study found that CSP is feasible and safe and improves LV function for HF patients with LVEF < 30%. Furthermore, CSP is associated with significantly improving clinical and echocardiographic outcomes, even for patients with non-CLBBB widened QRS.

### HF with severely reduced EF

Patients with HFsrEF are not uncommon in clinical practice and frequently present with advanced HF, particularly in elderly patients (11). Many patients have poor prognoses, persistent symptoms, and a high rehospitalization rate despite receiving optimal drug treatment. To the best of our knowledge, there is no consensus on the definition of severely reduced EF. LVEF ≤ 30% is a criterion for diagnosing advanced HF in ESC guidelines and is used as a severity partition cut-off value for 2-dimensional echocardiography-derived LVEF (12, 13). Our study's baseline LVEF of enrolled patients ranged from 15% to 27%, with considerably enlarged LVEDd and LVESV. The median baseline NHYA class was at grade 3.46, with 97.1% of patients in NHYA class above grade 3, demonstrating the severity of these patients.

Patients with HFsrEF are becoming more prevalent due to the ageing population, increasing number of HF patients, and improved treatment (3). A large study of patients with chronic HF demonstrated that improving LVEF was associated with better outcomes and a lower risk for cardiovascular events (14). However, although optimal medical therapies have been thoroughly studied to improve cardiac function, managing the severely reduced EF in patients with HF remains a therapeutic challenge. CSP may provide a practical treatment choice to improve clinical outcomes for these patients.

### CSP in patients with HFsrEF

To date, most guidelines recommend CRT in patients with LBBB and LVEF ≤ 35%, for those who subsequently develop symptomatic HF with decreased LV function following right ventricular (RV) pacing, and for atrioventricular (AV) block with poor LV function. CRT should also be considered for patients with AF who are candidates for AV node ablation, irrespective of QRS duration. CSP is an alternative strategy for achieving CRT. Several RCTs have demonstrated that CSP is superior to RV pacing in improving quality of life, NYHA class, and LV function in patients with HFrEF. However, the data on the benefit of CSP in patients with severely reduced EF was limited. The mean baseline LVEF values in several early small-sample studies focusing on HBP in HFrEF and LBB were less than 30% (15–17). The HBP was successful in 56%–76% of enrolled patients, achieving significant improvements in clinical outcomes and an approximately 5% increase in LVEF. Vijayaraman et al. evaluated His-Optimized CRT in 27 HF patients with a baseline LVEF of 24 ± 7% (NYHA class Ⅲ-Ⅳ). They reported a favorable clinical responder (NYHA class decreased to 2.04) in 84% and an echocardiographic response (LVEF grew to 38 ± 10%) in 92% of patients (18). Our study also demonstrated that CSP significantly improves HF symptoms, 6-MWD and NYHA classifications in patients with HFsrEF. Most patients had an echocardiographic response, and LV function improved significantly. Additionally, 77.1% of patients were CRT responders, 52.9% were CRT super-responder, and no patient had a reduction in LVEF during the follow-up. This satisfactory response rate to CSP provides us confidence in treating patients with HFsrEF with CSP.

Moreover, CSP has significantly facilitated implantation compared to CRT and has become more extensively employed in well-established centers. And, in our experience, with an experienced operator, CSP could be completed in a reasonably short time (in 2 h) and significantly decreased the duration of device implantation. Finally, even for patients with HFsrEF in the present study, none suffered from intraprocedural acute HF, and no severe complication was detected. Therefore, CSP may also be appropriate for patients with advanced HF.

In fact, CRT may be underutilized in these patients due to concerns about severe symptoms, a poor prognosis, and questionable improvement. Recently, a position statement on CRT by the Heart Failure Association (HFA) with several other European associations indicated that many HF patients are not exposed to the full potential benefit of CRT (19). They advocated for enhanced patient screening to identify potential eligible CRT candidates, ECG surveillance in HF patients, and comprehensive CRT education in primary and secondary care settings.

### CRT response in patients with HFsrEF

In our study, CRT response was observed in 77.1% of enrolled patients, indicating CSP was highly effective for patients with severely impaired systolic. On the other hand, the patients who had no response to CRT presented with a 2% increase in LVEF and a significant improvement in HF symptoms. In addition, symptom improvement was more pronounced and occurred earlier than echocardiographic improvement. This finding was consistent with most other studies, demonstrating that the proportion of super- and non-responders is low and that even non-responders are likely to obtain a clinical improvement (16, 20, 21). There is no consensus regarding the definition of CRT response or whether the echocardiographic response is a decisive criterion for CRT response (22). It is well known that LV reverse remodeling is poorly related to symptom improvement. LV remodeling is a long-term process and is likely to be maximal more than 2 years following CRT (23). Furthermore, patients with advanced decompensated HF who fail to achieve significant LV reverse remodeling in the early CRT post-implantation may still obtain hemodynamic benefits (24). Indeed, patients assessed as “non-responders” may have benefited symptomatically in NYHA class and 6-MWD. Thus, we believe that using echocardiographic response to evaluate CRT response may underestimate the benefits of CRT. HF is incurable, and the goal of treatment is to slow the disease progression. As CRT is a treatment for “disease modification”, the concept of “remission” and “non-remission” may be more appropriate for assessing the effect of CRT rather than “response” and “non-response” (19).

### Predictors of response to CRT

Several characteristics have been proven to predict a favorable CRT response. The patients with wide QRS and LBBB morphology, echocardiographic evidence of desynchrony, non-ischemic cardiomyopathy (ICM), and female sex responded favorably to CRT. In a meta-analysis of 5 randomized trials using individual patient data, QRS width was found to be a powerful predictor of response to CRT (25). In most guidelines, QRS duration ≥150 ms is listed as an inclusion criterion of recommendation for CRT. In contrast, CRT is not suggested in patients with a QRS duration <130 ms who are not candidates for RVP (26). Patients with typical LBBB QRS morphology show a better CRT response than patients with non-LBBB morphology (6). However, CRT response in patients with a non-CLBBB widened QRS remains uncertain. In our study, compared to patients with non-CLBBB, patients with CLBBB obtained more significant clinical and echocardiographic improvements from CSP.

The association between the etiology of HF and CRT response is also unclear. The magnitude of the echocardiographic response to CRT in patients with non-ICM is significantly higher than in those with ICM (27). Moreover, QRS narrowing after CRT was associated with clinical and echocardiographic CRT response (28). CRT response predictors are similar to those for reverse LV remodeling (29). A prospective registry study of outpatients with HFrEF found that shorter HF duration, no implantable cardioverter, lower LVEF, non-ICM, and no coronary disease were associated with significantly LVEF increase (30). However, only baseline BNP was associated with CRT response after multivariable analysis in our study.

This study has several limitations. First, this is a retrospective study with some patients without strict Strauss criteria for CLBBB. However, these patients represented only about 30% of this study and were compared with true CLBBB in terms of clinical outcomes. Moreover, most of the enrolled patients underwent the CSP procedure within the last 4 years. The CSP was performed mainly through one experienced operator, and only 4 patients were lost to follow-up, which helped to minimize bias. Second, the sample size was limited, but HF patients with LVEF < 30% who underwent CSP were relatively few. Third, the NYHA class is based on patients’ symptoms. It is worth noting that when a patient feels significantly better after CSP, their attention may be directed towards less frequent admission to the hospital. However, 6-MWD is an objective indicator of HF symptoms. Fourth, we could not completely exclude that pharmacological therapy for HF contributed to the favorable prognosis. However, due to the low blood pressure, most HF patients with very low EF are intolerant to these drugs. In our analysis, there was no difference in drug use between CSP responders and non-responders. Finally, as a retrospective study, this study lacked intraprocedural ECG data.

## Conclusions

CSP is feasible and safe in patients with HFsrEF, and it is associated with significantly better clinical and echocardiographic outcomes.

## Data Availability

The original contributions presented in the study are included in the article/**Supplementary Material**, further inquiries can be directed to the corresponding authors.
