# Supplementary material for: The feasibility and safety of his-purkinje conduction system pacing in patients with heart failure with severely reduced ejection fraction
Source: Front Cardiovasc Med. 2023 May 22;10:1187169. doi: 10.3389/fcvm.2023.1187169 (PMC10239933; doi:10.3389/fcvm.2023.1187169)
Supplement: Supplementary file 1 [file Table1.docx]

Supplementary Material

The Feasibility and Safety of His-Purkinje Conduction System Pacing in Patients with Heart Failure with Severely Reduced Ejection Fraction

Chengming Ma^1†^, Zhongzhen Wang^1†^, Zhulin Ma^2^, Peipei Ma^1^, Shiyu Dai^1^, Nan Wang^1^, Yiheng Yang^1^, Guocao Li^1^, Lianjun Gao^1^, Yunlong Xia^1^, Xianjie Xiao^1*^, Yingxue Dong^1*^

*** Correspondence:** Xianjie Xiao and Yingxue Dong: [dong_yignxue@126.com](mailto:dong_yignxue@126.com)

|  | CLBBB morphology (n=52) | non-CLBBB morphology (n=18) | *P* |
| --- | --- | --- | --- |
| Age | 66.4±9.15 | 66.83±7.99 | 0.52 |
| NYHA Class | 3.55±0.54 | 3.22±0.55 | 0.03 |
| 6-MWD | 157.98±53.23 | 189.7±52.62 | 0.032 |
| BNP | 812.98[401.92,2353.96] | 741.18[476.440,1143.4] | 0.427 |
| LVEDd | 68.6±7.0 | 63.7±7.8 | 0.015 |
| LVEF | 22.6±3.37 | 24.94±1.95 | 0.001 |
| LVESV | 218.5±37.8 | 178.38±33.81 | 0.007 |
| QRS duration | 168.22±21.99 | 139.67±43.33 | ＜0.001 |
| HF duration | 36 [12,96] | 5 [2,10] | ＜0.001 |

**Supplementary Table 1. Baseline clinical characteristics of CLBBB morphology and non-CLBBB morphology patients.**

**Supplementary Table 2. Postprocedural clinical outcomes of the patients and changes in TTE parameters.**

|  | CLBBB morphology (n=52) | non-CLBBB morphology (n=18) | *P* |
| --- | --- | --- | --- |
| NYHA Class | 1.88±0.94 | 1.83±0.86 | 0.839 |
| 6-MWD | 362.1±305.0 | 509.4±261.9 | 0.559 |
| LVEF | 35.04±10.86 | 34.61±8.98 | 0.881 |
| LVEDd | 61.4±8.91 | 61.39±9.14 | 0.995 |
| LVESV | 122.9±66.61 | 99.0±47.1 | 0.339 |
| QRS duration | 124.98±18.29 | 113.41±18.94 | 0.017 |
| ∆LVEF | 12.44±10.58 | 9.67±9.0 | 0.324 |
| ∆LVEDD | -7.19±8.0 | -2.28±4.93 | 0.004 |
| ∆LVESV | -95.6±52.81 | -79.38±45.85 | 0.421 |
| ∆LVESV/LVESV | 0.45±0.25 | 0.44±0.24 | 0.933 |
| ∆6-MWD | 304.13±290.9 | 319.7±257.5 | 0.841 |
| ∆QRS duration | -34.77±26.13 | 6.44±33.62 | ＜0.001 |
| Super-response | 59.6% | 33.3% | 0.054 |
| CRT response | 80.8% | 66.7% | 0.219 |
| HF Rehospitalizations | 0.5±0.9 | 0.5±0.9 | 0.869 |
| HF recovered months | 11.04±9.84 | 8.22±5.65 | 0.255 |
