# Supplementary figures and images for: The feasibility and safety of his-purkinje conduction system pacing in patients with heart failure with severely reduced ejection fraction
Source: Front Cardiovasc Med. 2023 May 22;10:1187169. doi: 10.3389/fcvm.2023.1187169 (PMC10239933; doi:10.3389/fcvm.2023.1187169)

## Slide 1
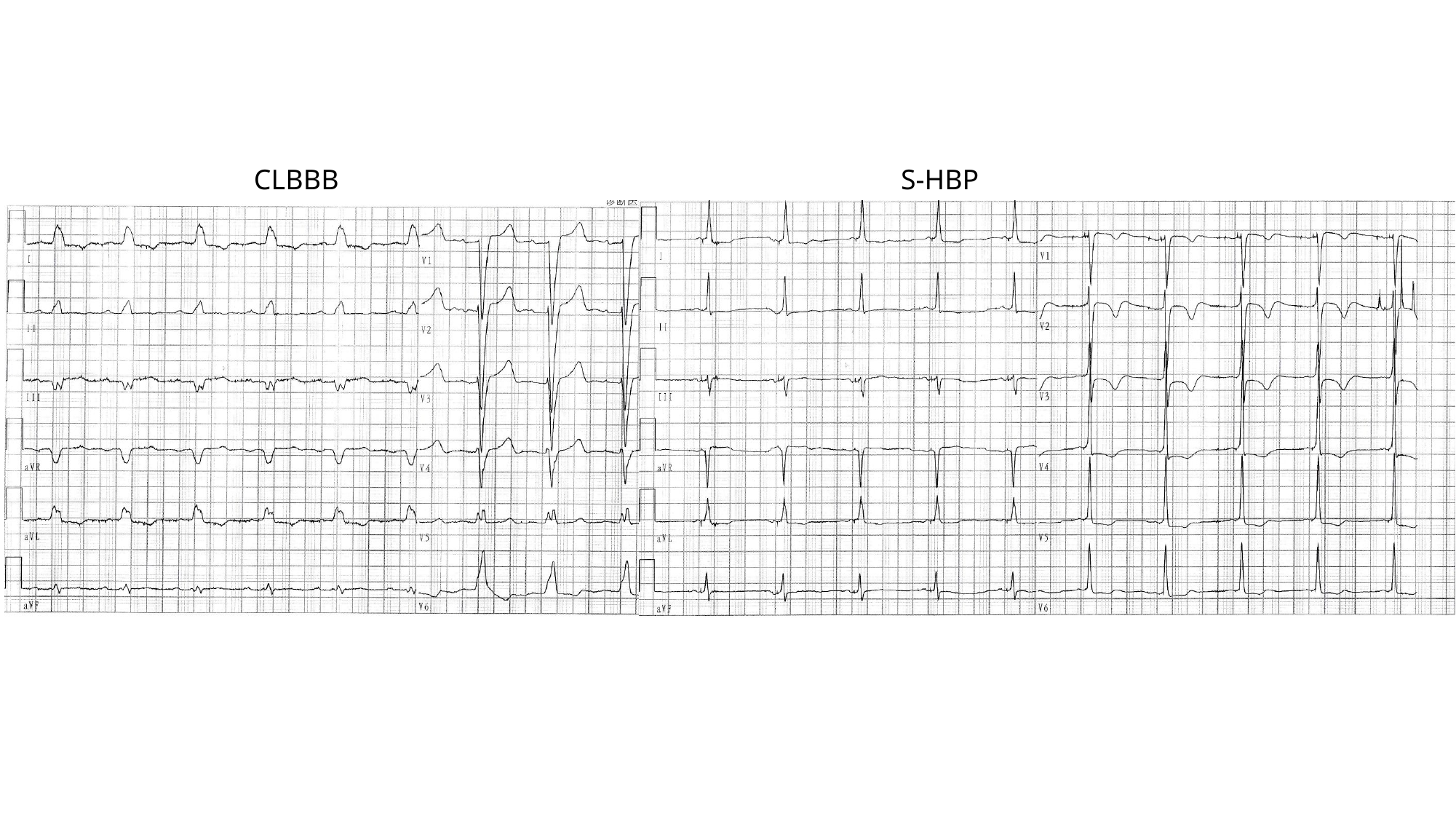

CLBBB
S-HBP

## Slide 2
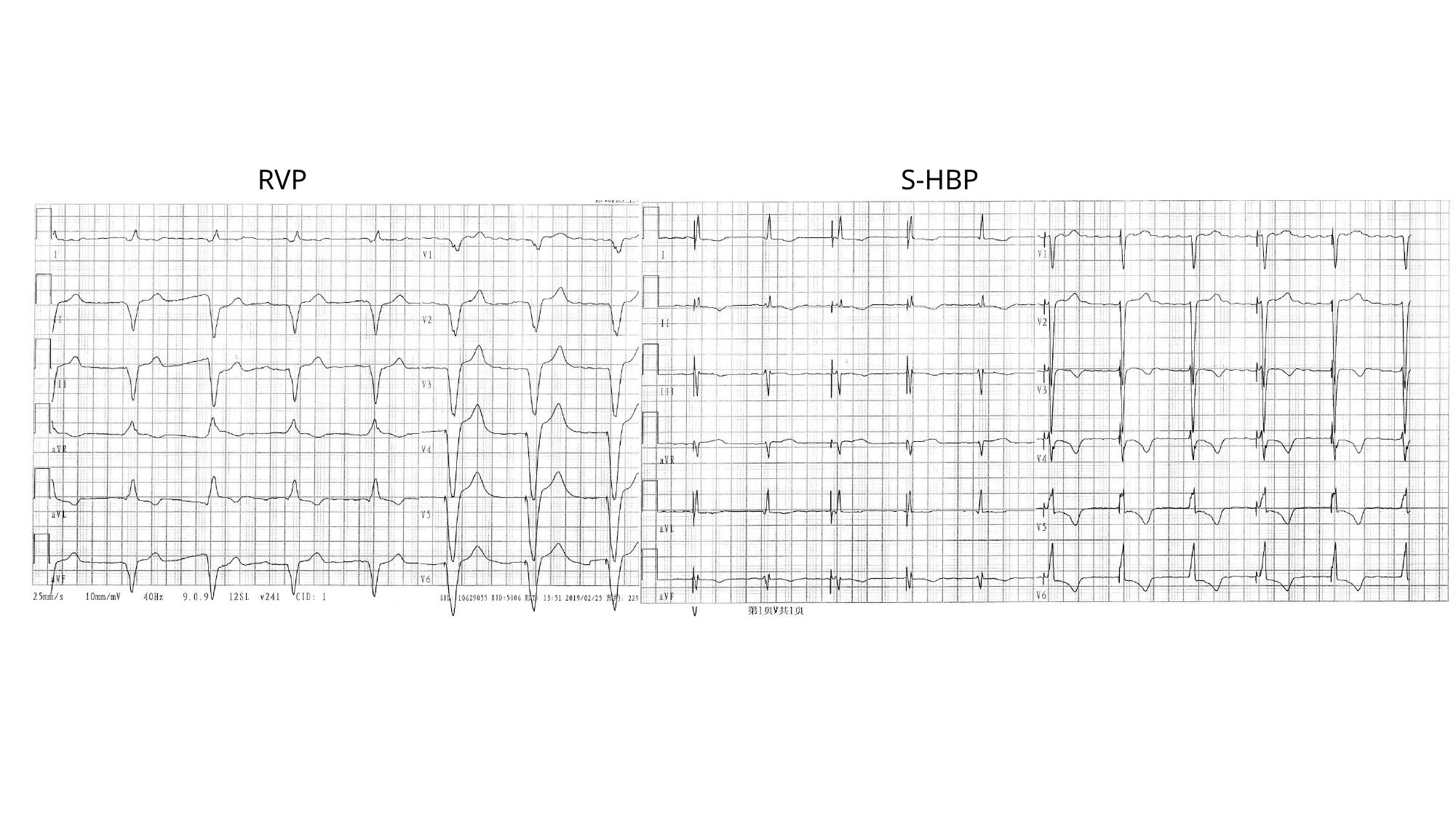

RVP
S-HBP

## Slide 3
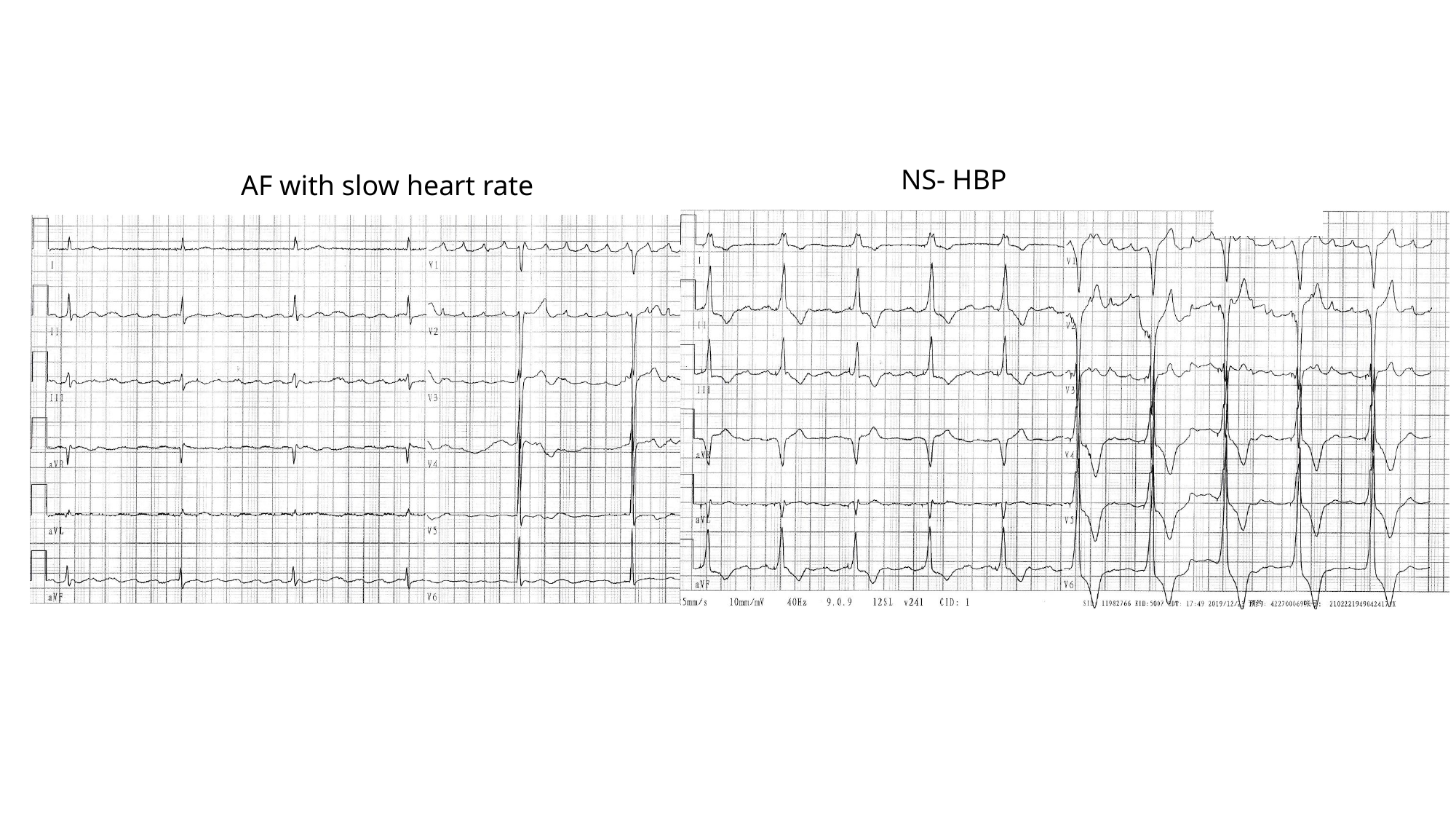

NS- HBP
AF with slow heart rate

## Slide 4
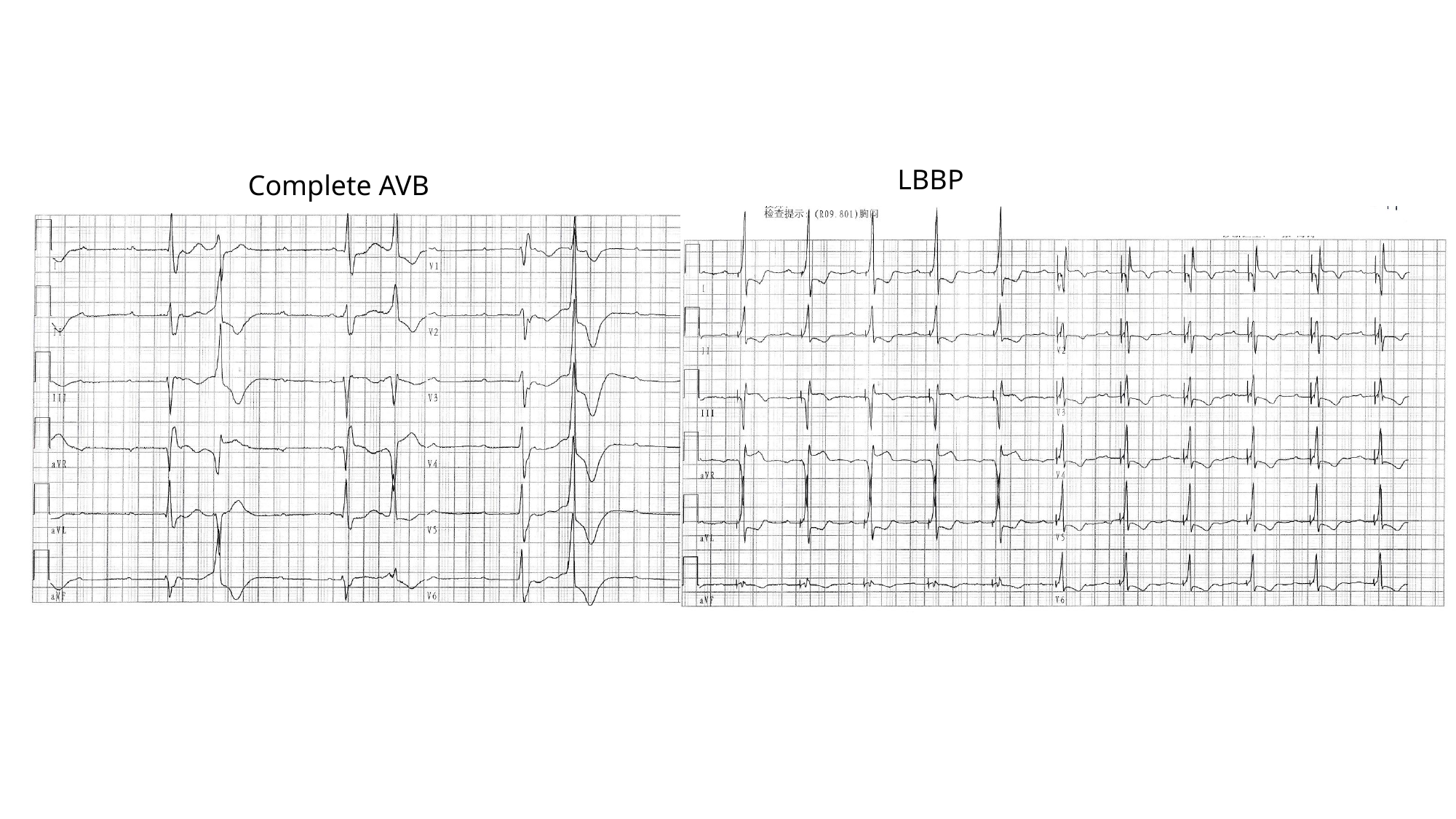

LBBP
Complete AVB

Supplement: Supplementary file 2 [file Presentation1.pptx]
